# Genomic relatedness of *Staphylococcus pettenkoferi* isolates of different origins

**DOI:** 10.1099/jmm.0.000472

**Published:** 2017-05-22

**Authors:** Emeli Månsson, Bengt Hellmark, Marc Stegger, Paal Skytt Andersen, Martin Sundqvist, Bo Söderquist

**Affiliations:** ^1^​ School of Medical Sciences, Faculty of Medicine and Health, Örebro University, SE-701 82 Örebro, Sweden; ^2^​ Centre for Clinical Research, Hospital of Västmanland Västerås, SE-721 89 Västerås, Sweden; ^3^​ Department of Laboratory Medicine, Faculty of Medicine and Health, Örebro University, SE-701 82 Örebro, Sweden; ^4^​ Statens Serum Institut, Artillerivej 5, 2300 Copenhagen S, Denmark

**Keywords:** *Staphylococcus pettenkoferi*, genotypic relatedness, repetitive-sequence based PCR typing, whole-genome sequencing

## Abstract

**Purpose:**

The aim of the study was to characterize clinical and environmental *Staphylococcus pettenkoferi* isolates with regard to genomic diversity and antibiotic susceptibility pattern. Repetitive-sequence-based PCR and core genome phylogenetic analysis of whole-genome sequencing (WGS) data verified the presence of distinct clades comprising closely related *S. pettenkoferi* isolates from different geographical locations and origins.

**Methodology:**

Phylogenetic relationships between 25 *S. pettenkoferi* isolates collected from blood cultures and intra-operative air sampling were determined by repetitive-sequence-based PCR typing and analysis of ~157 000 SNPs identified in the core genome after WGS. Antibiotic susceptibility testing and tests for biofilm production (microtitre plate assay) were performed.

**Results:**

Repetitive-sequence-based PCR as well as WGS data demonstrated the close relatedness of clinically significant blood culture isolates to probable contaminants, as well as to environmental isolates. Antibiotic-susceptibility testing demonstrated a low level of antimicrobial resistance. The *mecA* gene was present in two cefoxitin-resistant isolates. No isolates were found to produce biofilm.

**Conclusion:**

Close genomic relatedness of *S. pettenkoferi* isolates from different geographical locations and origins were found within clades, but with substantial genomic difference between the two major clades. The ecological niche of *S. pettenkoferi* remains unconfirmed, but the presence of *S. pettenkoferi* in the air of the operating field favours the suggestion of a role in skin flora. Identification of *S. pettenkoferi* in clinical samples should, in a majority of cases, most likely be regarded as a probable contamination, and its role as a possible pathogen in immunocompromised hosts remains to be clarified.

## Introduction

*Staphylococcus pettenkoferi* was first proposed as a novel staphylococcal species by Trülzsch *et al*. in 2002, after phenotypic and genotypic characterization of two clinical coagulase-negative staphylococci (CoNS) isolates from a German hospital [1]. In the past, *S. pettenkoferi* was probably often misidentified as other CoNS species, *Kocuria* sp. or *Micrococcus* sp. [1–3], because identification by biochemical methods such as analytical profile index (API) has been misleading and the species do not differ from other CoNS in terms of morphology or simple biochemical tests such as for oxidase, novobiocin or coagulase. With the introduction of MALDI-TOF mass spectrometry (MALDI-TOF MS) in clinical practice, species identification of *S. pettenkoferi* is no longer difficult, and hence correct identification of this novel staphylococcal species in clinical samples is likely to increase [4]. This was illustrated by a recent French study in which 54 of 9847 CoNS isolates were identified as *S. pettenkoferi* after implementation of MALDI-TOF MS, compared to 0 of 7885 CoNS isolates during a previous time period when phenotypic methods of identification were used [5].

*S. pettenkoferi* is believed to colonize human skin [4], but little is known about its ecological niche. According to 16S rRNA and *rpoB* sequencing, *S. pettenkoferi* is most closely related to *S. auricularis* [6]. Case reports of occasional clinical findings of *S. pettenkoferi* isolates include osteomyelitis, bloodstream infections, and bursitis [1–3, 7–10], predominantly in immunocompromised patients. The aim of the present study was to characterize clinical and environmental *S. pettenkoferi* isolates with regards to diversity and antibiotic susceptibility pattern.

## Methods

### Bacterial isolates

The clinical isolates (*n*=16) originated from blood cultures obtained at the Department of Clinical Microbiology, Central Hospital, Växjö, Sweden, in 2011–2013 (*n*=13; Vaxjo_1–13), at the Department of Clinical Microbiology, Örebro University Hospital, Örebro, Sweden in 2014 (*n*=1; Orebro_14), and as part of the European arm of the worldwide SENTRY Antimicrobial Surveillance Program during 2006 and 2008 (*n*=2; JMI_15–16; provided by JMI Laboratories, North Liberty, IA). The clinical isolates from Växjö and Örebro were provided to us together with clinical data noted on the original request form, and the sex and age of the patient, but identification of patients was not possible (Table 1). The microbiological relevance of the isolates was determined according to the European Manual of Clinical Microbiology criteria for interpretation [11]; that is, an isolate was considered a probable contaminant if only one blood culture bottle was positive.

**Table 1.** Characteristics of *S. pettenkoferi* isolates (*n*=25) obtained from blood cultures and air sampling in operating theatres during prosthetic joint surgery COPD, chronic obstructive pulmonary disease; CRP, C-reactive protein; TKA, total knee arthroplasty; THA, total hip arthroplasty. –, data not available.

Isolate ID	Year	Continent	Origin	Positive bottles/bottles cultured	Nosocomial	Clinical data	Age	Sex
Vaxjo_1	2011	Europe	Blood culture	1/4	No	Suspected sepsis	82	F
Vaxjo_2	2011	Europe	Blood culture	1/4	No	Elevated CRP	90	F
Vaxjo_3	2012	Europe	Blood culture	1/4	No	Elevated CRP, fever	56	M
Vaxjo_4	2012	Europe	Blood culture	1/4	Yes	Elevated CRP, shortness of breath	89	F
Vaxjo_5	2012	Europe	Blood culture	1/4	No	Suspected aspiration pneumonia	72	M
Vaxjo_6	2012	Europe	Blood culture	1/4	No	Fever, COPD	87	F
Vaxjo_7	2013	Europe	Blood culture	1/4	No	Suspected sepsis	60	M
Vaxjo_8	2013	Europe	Blood culture	–	–	–	–	–
Vaxjo_9	2013	Europe	Blood culture	2/4	No	Elevated CRP	85	M
Vaxjo_10	2013	Europe	Blood culture	1/4	Yes	Immunocompromised, fever	65	M
Vaxjo_11	2013	Europe	Blood culture	1/4	Yes	Fever, elevated CRP	50	M
Vaxjo_12	2013	Europe	Blood culture	1/4	No	Fever	16	F
Vaxjo_13	2013	Europe	Blood culture	1/4	Yes	Elevated CRP	83	F
Orebro_14	2014	Europe	Blood culture	2/4	No	Immunocompromised, fever	65	M
JMI_15	2006	North America	Blood culture	–	Yes	–	61	M
JMI_16	2008	Europe	Blood culture	–	No	–	–	–
**Isolate ID**	**Year**	**Continent**	**Origin**	**Surgery performed**	**Median c.f.u./m^2^**			
Vasteras_17 : 1–8	2011	Europe	Peri-operative air sampling	TKA	120			
Vasteras_18 : 1	2011	Europe	Peri-operative air sampling	THA	15			

The environmental isolates (*n*=9) originated from active air sampling during one prosthetic knee joint operation (*n*=8; Vasteras_17 : 1–8) and one prosthetic hip joint operation (*n*=1; Vasteras_18 : 1) using a Sartorius MD-8 air scanner (Sartorius Mechatronics, Göttingen, Germany) in the operating field at the Department of Orthopaedics, Central Hospital Västerås, Sweden, in November 2011, as described previously [12]. Repetitive-sequence-based PCR (rep-PCR) typing and phylogenetic relationship analysis based on whole-genome sequence (WGS) data, was performed with the inclusion of reference strain CCUG 51270 T (CCUG, Department of Clinical Bacteriology, University of Gothenburg, Sweden).

### Species identification

Species identification was performed using direct colony testing with MALDI-TOF MS (Microflex LT and Biotyper 3.1, Bruker Daltonics, Bremen, Germany). The cut-off for species identification was set at a score value ≥2.000. Species identification was verified by nucleotide sequence determination of a segment of the *rpoB* gene, performed as previously described for *S. epidermidis* [13].

### Rep-PCR typing

DNA from colonies was extracted using an UltraClean Microbial DNA isolation kit (bioMérieux, Marcy-l′Etoile, France) following the manufacturer's instructions. The extracted DNA was amplified using the DiversiLab *Staphylococcus* DNA fingerprinting kit (bioMérieux). The PCRs were performed on a thermal cycler (GeneAmp PCR System 9700, Applied Biosystem, Foster City, CA) with conditions including an initial pre-incubation at 94 °C for 2 min; 35 cycles of 94 °C for 30 s, 45 °C for 30 s, and 70 °C for 90 s; and a final extension at 70 °C for 3 min.

The amplified rep-PCR products were separated by electrophoresis performed in a microfluidics DNA LabChip (bioMérieux) and detected with an Agilent 2100 Bioanalyzer (Agilent Technologies, Palo Alto, CA).

Analysis was performed with version 3.6 of the DiversiLab software package (bioMérieux) using the Kullback–Leibler method to calculate similarity. The typing report provided by the software included a dendrogram with a virtual gel image of the samples and a scatter plot (a two-dimensional representation of relative sample similarity). Isolates were categorized as indistinguishable (>97 % similarity and no banding differences), similar (>95 % similarity and 1–2 band differences) or different (<95 % similarity and ≥2 band differences).

### Genome sequencing of *S. pettenkoferi* isolates

DNA from all isolates was extracted using a DNeasy Blood and Tissue kit as described by the manufacturer (Qiagen, Valencia, CA). For sequencing preparation on the MiSeq system, fragment libraries were constructed using the Nextera XT Kit (Illumina, San Diego, CA) followed by 251 bp paired-end sequencing on a MiSeq (Illumina) according to the manufacturer’s instructions. The sequencing reads were assembled using CLC Genomics Workbench 9.5 (Qiagen, Aarhus, Denmark) with default parameters to include only contigs ≥500 nt. The WGS data were aligned against the draft genome of *S. pettenkoferi* NZ_JVVL00000000.1 (strain 1286_SHAE) from the NCBI Reference Sequence Database (RefSeq) using the short-read alignment component of the Burrows–Wheeler aligner, as were two other available *S. pettenkoferi* draft genomes, NZ_JVAY00000000.1 (strain 589_SHAE) and NZ_AGUA00000000.1 (strain VCU012), from the RefSeq database using MUMmer. Each alignment was analysed for SNPs using NASP (https://github.com/TGenNorth/NASP), with exclusion of all SNPs that did not meet a minimum coverage of 10 or if the variant was present in ≥90 % of the base calls. SNPs identified in duplicated regions on the reference genome were removed. Phylogenetic relationships were reconstructed using the maximum-likelihood method implemented in PhyML (www.atgc-montpellier.fr/phyml/) using Smart Model Selection with 100 replicates on all included isolates. The Illumina data produced in this study were deposited in the European Nucleotide Archive under study IDs ERS1751294-ERS1751319.

### Antibiotic susceptibility testing

Isolates were tested for susceptibility be usinga standardized disk diffusion method as defined by the European Committee on Antimicrobial Susceptibility Testing (EUCAST) [14], using antibiotic disks (Oxoid, Basingstoke, UK), Mueller–Hinton II agar 3.8 % (w/v; BD Diagnostic Systems, Sparks, MD) and the following antibiotics: cefoxitin (30 µg), fusidic acid (10 µg), clindamycin (2 µg), erythromycin (15 µg), gentamicin (10 µg), rifampicin (5 µg), trimethoprim-sulfamethoxazole (25 µg) and norfloxacin (10 µg). Categorization of the isolates into Susceptible Intermediate Resistant (SIR) category was performed according to EUCAST clinical breakpoints (www.eucast.org).

### Detection of the *mecA* gene

All isolates were tested for presence of the *mecA* gene using a real-time PCR system (Light Cycler 2.0, Roche Diagnostics, GmbH, Mannheim, Germany) as previously described for *S. aureus* [15].

### Determination of biofilm production

A microtitre plate (MTP) assay was used for the detection of biofilm production, as described previously [13]. *S. epidermidis* RP62A was used as the positive control and *S. epidermidis* ATCC 12228 as the negative one.

## Results

Eleven *S. pettenkoferi* isolates retrieved from blood cultures were deemed probable contaminants, two (Vaxjo_9 and Orebro_14) were deemed clinically significant and data was not available for three isolates (Table 1).

### Genomic relatedness based on rep-PCR verified by core genome phylogenetic analysis of WGS data

**Fig. 1.**
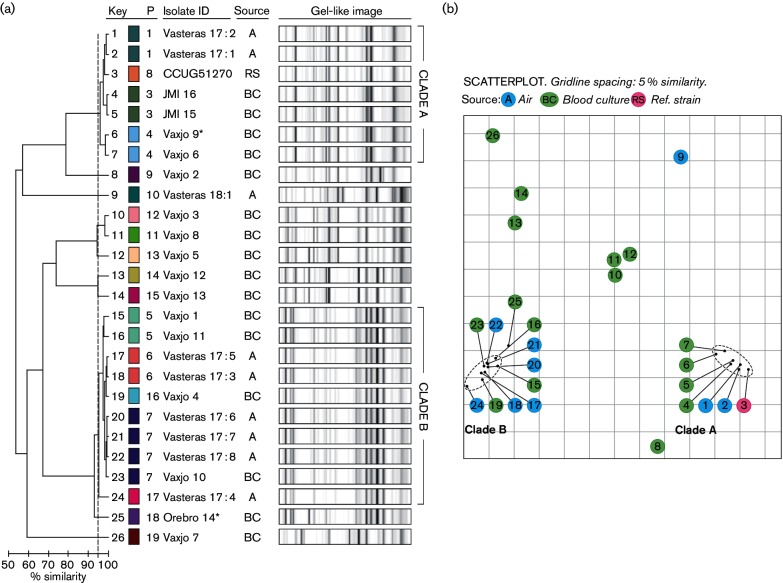
DiversiLab (DL) rep-PCR profiles of 26 *S. pettenkoferi* isolates. (a) Profiles and corresponding dendrogram obtained using the Kullback–Leibner method for correlation analysis. The similarity line (95 %) is presented as a light grey dotted vertical line. Each colour represents a single pattern (indistinguishable profiles). Isolates deemed clinically significant are marked with an asterisk. (b) Scatter plot of 26 *S. pettenkoferi* isolates. Each isolate is represented by its number corresponding to the DL profile, as presented in (a) ('key'). Colour represents origin of isolates [blue sampling (A); green, blood culture (BC); pinkreference strain (RS); pattern (P)].

**Fig. 2.**
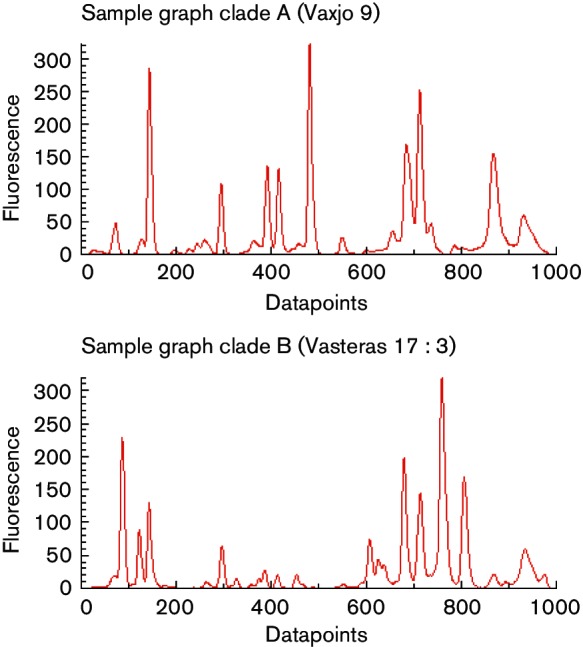
Examples of graphs of isolates belonging to clade A and B. Clade A is represented by the clinically significant isolate Vaxjo_9 and clade B is represented by the environmental isolate Vasteras 17 : 3.

Repetitive-sequence-based typing generated several high intensity peaks at various molecular weights for *S. pettenkoferi*. The dendrogram (Fig. 1a) and scatterplot (Fig. 1b) depicted two main clades (A and B) comprising 72 % of the isolates. The seven isolates in clade A included two indistinguishable clinical isolates from Europe, two indistinguishable environmental isolates from a prosthetic knee joint operation in Västerås, two indistinguishable clinical isolates from Växjö [including the clinically significant isolate Vaxjo_9, obtained from an 85-year-old man admitted to the emergency ward with worsening of general condition and elevated C-reactive protein (CRP)] and reference strain CCUG51270. Clade B included five environmental isolates and five clinical isolates (including the clinically significant isolate Orebro_14, obtained from a 75-year-old man with chronic lymphocytic leukaemia admitted to the infectious diseases ward due to neutropenic fever). The presence/absence and intensity of peaks differed between clade A and B, as illustrated in Fig. 2. The environmental isolates from operation 1 belonged to both clades, while the single environmental isolate from operation 2 had a unique rep-PCR fingerprint pattern.

**Fig. 3.**
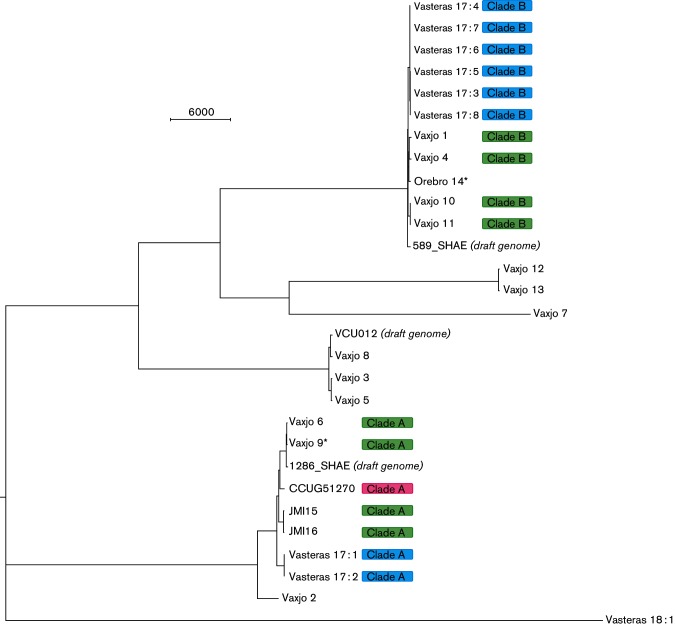
Midpoint-rooted maximum-likelihood approximation using FastTree 2.1.5 on the 157 438 core SNP within 26 isolates of *S. pettenkoferi* aligned with three available *S. pettenkoferi* draft genomes. Isolates deemed clinically significant are marked with an asterisk. Rep-PCR clade is presented to the right of isolate ID. Colour represents origin of isolates (blue, air sampling; green, blood culture; pink, reference strain). Scale bar indicates the number of SNP distances. Range of intra-clade SNP distances: clade A 11–1872, clade B 0–672.

In order to verify the findings of the DiversiLab rep-PCR typing, core genome phylogenetic analysis based on WGS data was performed. About 157 000 SNPs in the core of about 80 % of the selected reference were identified. The inferred relationship using WGS data was in agreement with the dendrogram obtained by the DiversiLab rep-PCR and demonstrated two major clades (A and B), which each comprised isolates that clustered by rep-PCR (Fig. 3). Two smaller clades were also in agreement with the rep-PCR dendrogram, as was the presence of two singletons (Vaxjo_7 and Vasteras_18 : 1). Intra-clade diversity was <2000 and <700 SNPs for A and B, respectively. By contrast, clade A and clade B differed by >58 000 SNPs from each other. Environmental isolate Vasteras_18 : 1, with a unique rep-PCR fingerprint pattern, differed from isolates in clade A and clade B with a minimum of 85 000 SNPs.

### Antibiotic susceptibility testing

Three clinical isolates (including one clinically significant isolate, Vaxjo_9), but no environmental isolates, were resistant to cefoxitin (Table 2). The *mecA* gene was detected by PCR in two of these cefoxitin-resistant isolates. The *mecC* gene was not detected in the third isolate by a specific *mecC* PCR (personal communication M. Bergman Jungeström, Linköping, Sweden) nor according to whole genome sequence data. Three isolates (12.5 %) were multidrug-resistant (i.e. resistant to ≥3 antimicrobial groups); two clinical isolates (both *mecA*-positive) and one environmental isolate. All isolates were susceptible to rifampicin and gentamicin.

**Table 2.** Antibiotic susceptibility patterns of *S. pettenkoferi* isolates (*n*=25) Antimicrobial susceptibility testing according to EUCAST guidelines. S, susceptible (white); R, resistant (light grey).

Isolate ID	Year	Cefoxitin	Fusidic acid	Clindamycin	Erythromycin	Gentamicin	Rifampicin	TMP/SMX	Norfloxacin	
Vaxjo_1	2011	R	S	S	S	S	S	S	S	
Vaxjo_2	2011	S	S	S	S	S	S	S	S	
Vaxjo_3	2012	S	R	S	S	S	S	S	S	
Vaxjo_4	2012	S	S	S	S	S	S	S	S	
Vaxjo_5	2012	S	S	S	S	S	S	R	S	
Vaxjo_6	2012	R*	R	R	R	S	S	S	R	
Vaxjo_7	2013	S	S	S	R	S	S	S	S	
Vaxjo_8	2013	S	R	S	S	S	S	S	S	
Vaxjo_9	2013	R*	S	R	R	S	S	S	R	
Vaxjo_10	2013	S	S	S	S	S	S	S	S	
Vaxjo_11	2013	S	S	S	S	S	S	S	S	
Vaxjo_12	2013	S	S	S	S	S	S	S	S	
Vaxjo_13	2013	S	S	S	S	S	S	S	S	
Orebro_14	2014	S	S	S	S	S	S	S	S	
JMI_15	2006	S	S	S	S	S	S	S	S	
JMI_16	2008	S	S	S	S	S	S	S	S	
Vasteras_17 : 1	2011	S	R	S	S	S	S	S	S	
Vasteras_17 : 2	2011	S	R	R	R	S	S	S	S	
Vasteras_17 : 3	2011	S	S	S	S	S	S	S	S	
Vasteras_17 : 4	2011	S	S	S	S	S	S	S	S	
Vasteras_17 : 5	2011	S	S	S	S	S	S	S	S	
Vasteras_17 : 6	2011	S	S	S	S	S	S	S	S	
Vasteras_17 : 7	2011	S	S	S	S	S	S	S	S	
Vasteras_17 : 8	2011	S	S	S	S	S	S	S	S	
Vasteras_18 : 1	2011	S	S	S	S	S	S	S	S	

*Isolate *mecA*-positive.

### Biofilm production

None of the clinical or environmental *S. pettenkoferi* isolates, nor the reference strain, were found to be biofilm-producing by the MTP assay.

## Discussion

The aim of the study was to characterize clinical and environmental *S. pettenkoferi* isolates with regard to diversity and antibiotic susceptibility pattern. Rep-PCR and core genome phylogenetic analysis obtained by WGS data verified the presence of distinct clades, each comprising closely related *S. pettenkoferi* isolates from different geographical locations and origins. Substantial genomic differences were observed between the two major clades, but the overall resemblance to the reference chromosome of *S. pettenkoferi* in the NCBI Reference Sequence Database was high (~87 %, data not shown).

In this study, *S. pettenkoferi* was regarded as a probable pathogen in the minority of patients with positive cultures. This is in accordance with previous studies. Argemi *et al.* [5] found that 45 of 50 *S.* *pettenkoferi* isolates obtained from various cultures could be determined as not microbiologically relevant and only one isolate was regarded as clinically relevant. In another study from Korea [10], where six blood culture isolates of *S. pettenkoferi* were characterized, S*. pettenkoferi* was interpreted as a probable pathogen in only one case (septic shock and aspiration pneumonia in a patient with chronic alcohol abuse and diabetes mellitus). The other five findings were categorized either as contaminants or as colonization of indwelling catheters. Consequently, although the pathogenic potential of *S. pettenkoferi* may not yet be fully understood, it is likely to be low.

The clinically significant isolates in this study were closely related, by rep-PCR and analysis of WGS data, to isolates regarded as contaminants. This may indicate that they represent clones present as part of the normal flora on the skin. In the present study, several of the blood culture isolates deemed as contaminants were nosocomial. Genetically diverse *S. pettenkoferi* isolates were retrieved from intra-operative air sampling: two isolates (Vasteras_17 : 1–2) were closely related to the reference strain CCUG 51270 T and three isolates (Vasteras_17 : 6–8) were indistinguishable from a blood culture isolate (Vaxjo_10) from a different hospital and year, according to rep-PCR. Nosocomial spread of *S. pettenkoferi* has previously been reported from a French ICU [7].

*Staphylococcus* spp. preferably colonize moist areas of the skin [16], but the ecological niche of *S. pettenkoferi* remains to be clarified. The genetically related *S. auricularis*, which is believed to have the external auditory canal as its principal habitat [4], was not encountered during air sampling in operating theatres [12], which might have been expected if these species share habitats. In a study in a small animal clinic, aiming to investigate the potential transmission of methicillin-resistant staphylococci, two multidrug-resistant isolates of *S. pettenkoferi* were isolated from swabs taken from a cat cage, but none were found in swabs taken from pets (*n*=10) or employees (*n*=4) [17]. *S. pettenkoferi* has also been isolated from sampling of public restrooms in London [18]. Since *S. pettenkoferi* occurs as a rare contaminant of blood cultures obtained via puncture of the skin as well as in the air in operating theatres during surgery, it is probably part of the normal flora of the skin, at least in some individuals. However, an environmental source may also be possible.

Previous reports of clinical findings of *S. pettenkoferi* include a limited number of cases of osteomyelitis, bloodstream infections in immunocompromised hosts and bursitis [1–3, 7–10, 19]. *S. pettenkoferi* has not yet been reported to be associated with implant infections and then *S. pettenkoferi* isolates included in this study did not form biofilm phenotypically. Variable antibiotic susceptibility profiles for *S. pettenkoferi* have been reported. In a collection of 44 isolates, 43.2 % were resistant to oxacillin [5], but the reported frequency of resistance to oxacillin varies between 12.5 % (present study; cefoxitin screen) and 83 % [10]. In the present study, antimicrobial resistance of *S. pettenkoferi* was low, with only a few multidrug-resistant isolates found.

A limitation of this study is that it includes a relatively small number of isolates that were almost all obtained from the same country. However, the isolates originated from different counties, and comprised both clinical and environmental samples. To our knowledge, this is the first study of the genetic diversity of *S. pettenkoferi* isolates.

To conclude, in this study, *S. pettenkoferi* isolated from different geographical locations and origins demonstrated close genomic relatedness within clades, but with substantial genomic differences between the major clades. Isolates interpreted as clinically significant were closely related to environmental isolates, irrespective of geographic origin, as well as to clinical isolates regarded as contaminants. Identification of *S. pettenkoferi* in clinical samples should, in a majority of cases, most likely be regarded as a probable contamination, and its role as a possible pathogen in immunocompromised hosts remains to be clarified.
